# Comprehensive Analysis Reveals Novel Interactions between Circulating MicroRNAs and Gut Microbiota Composition in Human Obesity

**DOI:** 10.3390/ijms21249509

**Published:** 2020-12-14

**Authors:** Taís Silveira Assmann, Amanda Cuevas-Sierra, José Ignacio Riezu-Boj, Fermín I. Milagro, J. Alfredo Martínez

**Affiliations:** 1 Center for Nutrition Research, Department of Nutrition, Food Science and Physiology, University of Navarra, Irunlarrea 1, 31008 Pamplona, Spain; taisassmann@hotmail.com (T.S.A.); acuevas.1@alumni.unav.es (A.C.-S.); jiriezu@unav.es (J.I.R.-B.); jalfmtz@unav.es (J.A.M.); 2 Centro de Investigación Biomédica en Red de la Fisiopatología de la Obesidad y Nutrición (CIBERobn), Carlos III Health Institute, 28029 Madrid, Spain; 3 IdiSNA—Navarra Institute for Health Research, 31008 Pamplona, Spain

**Keywords:** BMI, miRNA, obesity, *Bacteroides eggerthi*, gut microbiota

## Abstract

Background: The determinants that mediate the interactions between microRNAs and the gut microbiome impacting on obesity are scarcely understood. Thus, the aim of this study was to investigate possible interactions between circulating microRNAs and gut microbiota composition in obesity. Method: The sample comprised 78 subjects with obesity (cases, body mass index (BMI): 30–40 kg/m^2^) and 25 eutrophic individuals (controls, BMI ≤ 25 kg/m^2^). The expression of 96 microRNAs was investigated in plasma of all individuals using miRCURY LNA miRNA Custom PCR Panels. Bacterial DNA sequencing was performed following the Illumina 16S protocol. The FDR correction was used for multiple comparison analyses. Results: A total of 26 circulating microRNAs and 12 bacterial species were found differentially expressed between cases and controls. Interestingly, an interaction among three miRNAs (miR-130b-3p, miR-185-5p and miR-21-5p) with *Bacteroides eggerthi* and BMI levels was evidenced (r^2^ = 0.148, *p* = 0.004). Moreover, these microRNAs regulate genes that participate in metabolism-related pathways, including fatty acid degradation, insulin signaling and glycerolipid metabolism. Conclusions: This study characterized an interaction between the abundance of 4 bacterial species and 14 circulating microRNAs in relation to obesity. Moreover, the current study also suggests that miRNAs may serve as a communication mechanism between the gut microbiome and human hosts.

## 1. Background

Obesity is a worldwide epidemic that arises as a chronic long-term imbalance between calorie intake and energy expenditure [1]. Despite nutritional interventions and physical education programs, the prevalence of obesity is still increasing and ∼600 million people worldwide are expected to have obesity by 2025 [2]. Obesity is the result of complex and not completely understood pathological processes deriving from a crosstalk among environmental factors, genetic susceptibility and epigenetic mechanisms [3,4].

Among the epigenetic mechanisms, microRNAs (miRNAs) are a class of small non-coding RNAs that regulate gene expression [5,6,7]. These molecules have recognized roles in the regulation of several biological processes, including cell cycle, cellular differentiation, proliferation, metabolism, ageing and apoptosis [7]. Moreover, it is estimated that miRNAs regulate the expression of more than 60% of protein-coding genes [5]; and, consequently, changes in their expressions and functions have been linked to many diseases, including metabolic disorders and obesity [8,9].

Recent findings indicate that host miRNAs contribute to the regulation of gut microbiome, specially involving at least two main processes: (i) host-secreted miRNAs regulate the gut microbiota; and (ii) the gut microbiota affects the host through inducing special miRNA expression [10]. Indeed, evidences suggest that miRNAs produced by host’s intestinal epithelial cells (IECs) participate in shaping the gut microbiota and affect bacterial growth [11]. These miRNAs target bacterial mRNA and then the host controls the gut microbiota via bacterial mRNA degradation or translational inhibition [11]. On the other hand, it was demonstrated, using *Dicer1* knock-out mice, that miRNAs were essential for epithelial cell proliferation, differentiation, nutrient absorption and that defective miRNA biogenesis was also responsible for impaired intestinal barrier function [12].

Additionally, gut microbiota regulates miRNA expression in IECs subtypes and this regulation may alter intestinal homeostasis [13]. In this sense, it was demonstrated that the expression of some miRNAs is different among IEC subtypes and the difference depends on microbial patterns [14]. Similarly, the expression of 16 miRNAs was found altered in the caecum of conventionally raised vs. germ-free mice [15]. Recently, it has been reported that gut microbiota specifically controlled the miR-181 family expression in white adipocytes during homeostasis to modulate key pathways related to adiposity, insulin sensitivity and white adipose tissue (WAT) inflammation in mice [16]. Furthermore, high-fat diet (HFD) feeding altered the composition of gut microbiota, leading to aberrant overexpression of miR-181 in WAT adipocytes [16]. Altogether, these studies provide clues that gut microbiota regulates host gene expression through modulation of host miRNA signature and that host metabolism could be influenced by this interaction.

According to these findings, miRNAs appear to play an important role in host-to-microbe interaction and may be considered as molecular targets for novel anti-microbial therapies to be developed. However, very little is known about the interactions between miRNAs and the host microbiome in the context of obesity. Therefore, the aim of this study was to investigate interactions between circulating miRNA patterns and gut microbiota composition in obesity.

## 2. Results

### 2.1. Clinical and Laboratory Characteristics of Individuals Included in the Study

Clinical, laboratorial and nutritional characteristics of cases with obesity and normal weight controls are shown in Table 1. There were no differences between cases and controls regarding age, gender, smoking habits, alcohol consumption, and energy intake. Moreover, both groups had a comparable dietary macronutrient composition. As expected, subjects with obesity presented higher waist circumference, glucose, total cholesterol, and triglyceride levels compared to normal weight individuals. Additionally, cases also presented elevated levels of metabolic markers such as insulin, leptin, Triglycerides-Glucose (TyG) and HOMA-IR indexes and, lower levels of metabolic equivalents (METs) compared to controls.

### 2.2. Quality Control of MicroRNA Expression

The RNA spike-in expressions presented low variation in Cq among samples in RNA isolation and cDNA synthesis, demonstrating that extraction, reverse transcription, and qPCR were effective and none of the samples contained inhibitors. As expected, the expression of UniSp2, UniSp4, UniSp5 and UniSp6 did not differ between groups (cases vs. controls). UniSp5 was expressed in all analyzed samples, demonstrating that miRNAs expressed at low levels was not lost during isolation. Thus, the mean cycle of each spike-in was calculated for the whole sample as follows: UniSp2: 20.4 ± 1.2, UniSp3: 22.5 ± 0.4, UniSp4: 27.1 ± 1.1, UniSp5: 34.2 ± 1.3 and UniSp6 19.4 ± 1.1. The ratio between miR-451a and miR-23a-3p ranged between 5 and −1, indicating that the samples were not affected by hemolysis. Generally, these results showed a good and similar level of sample quality and reproducibility of the miRNA profiling processes.

### 2.3. MicroRNAs Differentially Expressed in Plasma of Patients with Obesity

The expression of 86 target miRNAs was evaluated in plasma of subjects with obesity and in normal weight individuals. Of these 86 miRNAs, 61 were expressed in at least 20% of the sample with Cq values ≥ 35. Of these 61 miRNAs, 26 were differentially expressed between cases and controls after FDR correction (Table 2). The results remained significant after adjustment for age and gender (Table 2 and Table S2). In addition, after adjustment for gender, triglycerides levels, and HOMA-IR index, 24 miRNAs remained associated with obesity (Table 2 and Table S2).

**Figure 1:**
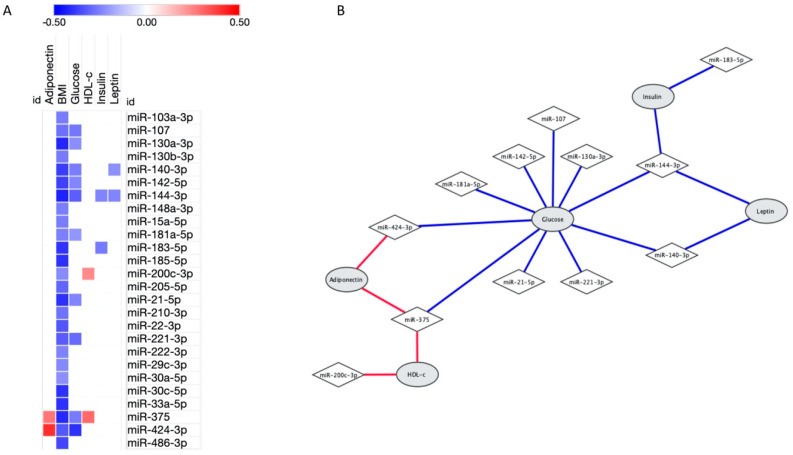
Significant correlations between 26 microRNAs associated with obesity and laboratorial characteristics. (**A**) Heatmap showing the correlations between microRNAs expression (in rows) and characteristics (in columns). Positive correlations were highlighted in red, negative correlations in blue and lack of correlation in white; brighter shades indicate higher correlations. (**B**) Network demonstrating correlation between microRNAs and laboratorial characteristics. Of note, all microRNAs also correlated with body mass index (BMI). Correlations analysis were performed using Pearson.

As expected, all these 26 miRNAs were negatively correlated with body mass index (BMI, *p* ≤ 0.05, Figure 1A). Moreover, as shown in Figure 1B, miR-107, miR-130a-3p, miR-140-3p, miR-142-5p, miR-144-3p, miR-181a-5p, miR-21-5p, miR-221-3p, miR-375 and miR-424-3p expressions were negatively associated with glucose levels (*p* < 0.05). Otherwise, miR-200c-3p and miR-375 positively correlated with HDL-c levels (r *=* 0.232, *p* = 0.047; and r *=* 0.295, *p* = 0.015, respectively). Regarding hormone levels, miR-140-3p and miR-144-3p were negatively associated with leptin levels (r *=* −0.222, *p* = 0.034; and r *=* −0.245, *p* = 0.025, respectively), miR-144-3p and miR-183-5p were inversely correlated with insulin (r *=* −0.245, *p* = 0.032; and r *=* −0.264, *p* = 0.033, respectively) and adiponectin levels were positively associated with miR-375 (r *=* 0.272, *p* = 0.025) and miR-424-3p (r *=* 0.405, *p* = 0.012).

### 2.4. Gut Microbiota Profile in Subjects with Obesity Compared to Eutrophic Individuals

**Figure 2:**
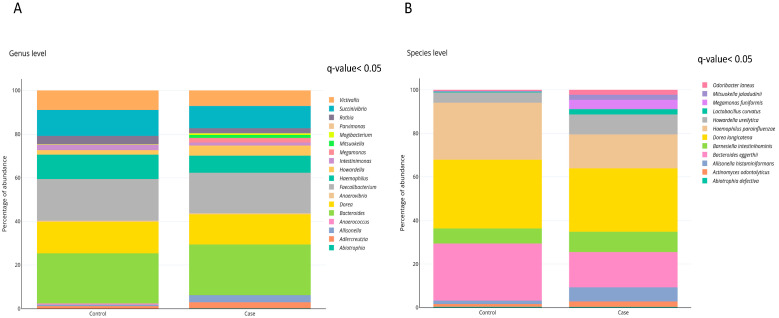
Gut microbiota composition in subjects with and without obesity. Relative abundance of (**A**) Bacterial genera. (**B**) Bacterial species. Differences in bacterial relative abundance at the genus and species levels in cases with obesity and controls with normal weight. Only genera or species whose abundances were significantly different (FDR, *q*-value < 0.05) are shown. *p*-values were obtained using Student *t*-test with the log-transformed variable and raw *p*-values were corrected by false discovery rate (FDR; *q*-value). The percentage of occurrence of each taxon is reported as cumulative bar chart. This figure was performed using ploty.com.

The effect of obesity on gut microbiota composition was investigated at the genus and species levels. The levels of eighteen bacterial genera were significantly different when comparing obese and normal weight individuals, being nine bacterial genera significantly increased in obese subjects when compared to controls (Figure 2A). Twelve bacterial species were statistically different between obese and normal weight individuals, being ten of them more abundant in subjects with obesity compared to eutrophic individuals (Figure 2B and Table 3). After adjustment for model 1 (age and gender), nine bacterial species remained associated with obesity (Table 3 and Table S3). In addition, after further adjustments for model 2 (gender, triglycerides levels and HOMA-IR index), seven bacterial species remained associated with obesity (Table 3 and Table S3). Of note, because of the very low abundance of *Abiotrophia defectiva* and *Mitsuokella jaladudinii,* the regression models analyses were not performed.

Shannon index, which reflects the alpha diversity, was not different between obese and normal weight groups (Figure S1). However, the beta diversity values of gut microbiota, based on Jaccard index (PERMANOVA, *p* = 0.025; Figure S2A) and Bray-Curtis dissimilarity (PERMANOVA, *p* = 0.015; Figure S2B), was significantly different between groups.

### 2.5. Crosstalk between Host MiRNAs and Gut Microbiota

**Figure 3:**
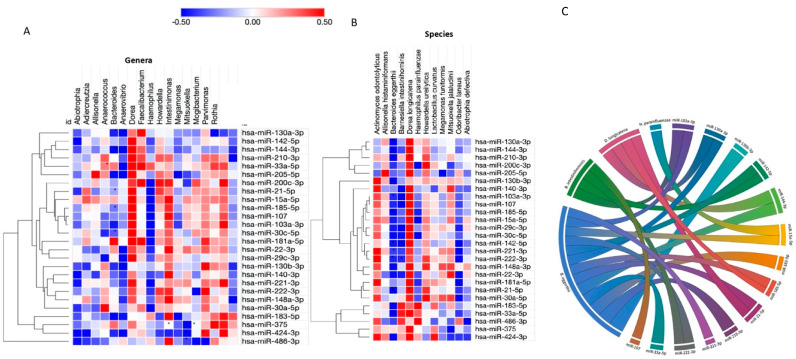
Correlation between miRNA and gut microbiota (genera and species) significantly associated with obesity. (**A**) Heatmap showing the correlations between bacterial genera (in columns) and differently expressed miRNAs (in rows). (**B**) Heatmap showing the correlation between bacterial species (in columns) and differently expressed miRNAs (in rows). MiRNAs were clustered using the one minus Pearson correlation with average linkage. Positive correlations were shown in red and negative correlations in blue, with brighter shades indicating higher correlations. Lack of correlations is represented in white. Statistically significant correlations were marked with asterisks (*). (**C**) Chord diagram demonstrating interaction between microRNAs and bacterial species. This figure was performed using MORPHEUS and ploty.com.

To further investigate the relationships between circulating miRNAs and the gut microbiota composition, associations between bacteria and miRNAs differentially expressed in obesity were analyzed. At the genus level, of the 18 genera differently expressed in obesity, 9 were significantly correlated with the expression of 10 miRNAs out of 26 miRNAs differently expressed in subjects with obesity (Figure 3A). Fourteen of these miRNAs were significantly associated with four bacterial species (*Dorea longicatena, Banesiela intestinihominis, Bacteroides eggerthii and Haemophillus parainfluenzae*), as illustrated in Figure 3B,C.

A diagram was built to visualize the relationships between the miRNAs and their significantly correlated bacteria (Figure 3C). The correlation network shows a highly interconnected relationship between these miRNAs and bacterial species. Interestingly, *B. eggerthii* negatively correlated with miR-103a-3p, miR-21-5p, miR-130a-3p, miR-185-5p, miR-144-3p, miR-210-3p, miR-33a-5p, miR-15a-5p, miR-130b-3p, miR-183-5p, miR-221-3p, miR-222-3p and miR-142-5p. Moreover, an interaction among miRNAs, *B. eggerthi* and BMI levels was found. Individually, the expression of miR-103a-3p (r^2^ *=* 0.1229, *p* = 0.051), miR-130b-3p (r^2^ *=* 0.0933, *p* = 0.021), miR-185-5p (r^2^ *=* 0.0894, *p* = 0.035), miR-21-5p (r^2^ *=* 0.1124, *p* = 0.008) and miR-210 (r^2^ *=* 0.0866, *p* = 0.052) interacted with these bacterial species and BMI levels. Furthermore, an interaction among three miRNAs levels (miR-130b-3p, miR-185-5p and miR-21-5p), *B. eggerthi* and BMI levels was also evidenced (r^2^ *=* 0.148, *p* = 0.004). Interestingly, there was also an interaction among *B. eggerthi*, adiponectin levels and miR-183-5p (r^2^ *=* 0.1294, *p* = 0.009).

In the same way, *B. intestinihominis* abundance was negatively associated with miR-107, miR-103a-3p, miR-222-3p and miR-142-5p expressions. The expression of miR-15a-5p was inversely associated with the abundance of *H. parainfluenzae* and an interaction with insulin levels (r^2^ *=* 0.0592, *p* = 0.027) was found. In contrast, *D. longicatena* was positively associated with miR-21-5p, miR-130a-3p, miR-185-5p and miR-144-3p. However, interactions among the bacterial abundance, miRNA expression and BMI levels were not found for these three bacterial species. No association among the bacterial species, miRNAs and leptin was found.

### 2.6. Predicted Functions of MiRNAs Correlated with Obesity-Associated Bacteria

Target gene prediction of the 14 miRNAs that correlated with the 4 bacterial species associated with obesity was accomplished using distinct databases of miRNA-gene interaction in miRWalk environment (Table S4). Of the 9584 genes identified as potential targets of these miRNAs, 5381 were found to be regulated by two or more miRNAs (Table S4); however only 719 genes were previously experimentally validated, according to miRTarBase (Figure S1). After that, functional enrichment analysis of miRNA targets was carried out to explore biological pathways possibly regulated by this set of miRNAs using KEGG Pathways via PathDIP website. A total of 248 KEGG pathways were significantly enriched (*q*-value < 0.05) for these miRNAs (Table S5). However, considering only the experimentally validated target genes, 98 pathways were significantly enriched (Table S5).

**Figure 4:**
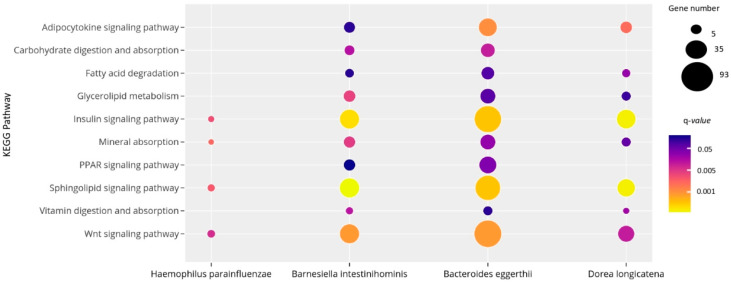
Significant KEGG pathways regulated by the 14 miRNAs correlated with bacteria differentially expressed in subjects with obesity. The size and the color of the dots represent the gene number and the range of the pathway’s *q*-value, respectively. The *y*-axis represents the KEGG pathways and the *x*-axis shows the 4 bacterial species associated with each selected pathway. *q*-values: *p*-values corrected for multiple tests using the Benjamini–Hochberg method. This figure was performed using ploty.com.

As shown in Figure 4, *H. parainfluenzae, D. longicatena, B. intestinihominis and B. eggerthii* correlated with miRNAs associated with pathways related to obesity and metabolic processes, including carbohydrate and lipid turnover, endocrine and inflammatory signaling pathways. More specifically, the target genes of miRNAs associated with the four bacterial species related to obesity participate in the fatty acid degradation, mineral absorption, carbohydrate digestion and absorption, insulin signaling pathway and glycerolipid metabolism.

## 3. Discussion

Over the past decade, there has been an increasing attention about the crucial roles played by miRNAs in a wide variety of cellular processes [9]. In the present study, 26 miRNAs were found as differentially expressed in plasma of subjects with obesity compared to normal weight individuals. Furthermore, the expression of 14 miRNAs (miR-107, miR-103a-3p, miR-142-5p, miR-222-3p, miR-221-3p, miR-183-5p, miR-183-5p, miR-130b-3p, miR-15a-5p, miR-33a-5p, miR-210-3p, miR-144-3p, miR-185-5p, miR-130a-3p and miR-21-5p) was linked with the relative abundance of 4 bacterial species that also significantly differed between cases and controls (*D. longicatena, B. intestinihominis, B. eggerthii* and *H. parainfluenzae*).

These miRNAs that interacted with obesity-associated bacteria regulate the expression of genes that participate in several metabolism and obesity-related pathways, such as carbohydrate and lipid metabolism, endocrine and inflammatory signaling pathways. Indeed, evidence suggests that the majority of miRNAs do not regulate a specific or individual target gene but rather they modulate the expression of large number of genes in networks, demonstrating their importance in the regulation of several metabolic processes [7,16].

Moreover, compelling evidence suggests that circulating miRNAs are associated with obesity [17,18,19,20]. Some miRNAs have been implicated in the control of body weight gain, glucose homeostasis, insulin resistance and lipid metabolism [21,22]. In this context, some of miRNAs associated with obesity in our study were also described in a recently systematic review study about obesity [23]. In agreement with our results, miR-21-5p and miR-103a, miR-221-3p were also found as downregulated in blood sample of subjects with obesity in a meta-analysis study [24]. Additionally, the miRNAs that were dysregulated in obesity in the present study are associated with various metabolic processes, such as impaired glucose intolerance [18], maintenance of the pancreatic beta cell mass [25], adipocyte development and adipose tissue physiology [8,26], inflammation pathways [27] and cardiomyocyte survival [28].

Additionally, an interaction between BMI levels, *B. eggerthii* abundance and the expression of three miRNAs (miR-130b-3p, miR-185-5p and miR-21-5p) was also evidenced. Interestingly, *B. eggerthii* is one of the intestinal bacteria that metabolize phenolic acids, which are regarded as beneficial for human health [29]. In a recent study, *B. eggerthii* abundance was significantly higher in children with obesity and positively correlated with body fat percentage but negatively with insoluble fiber intake in Mexican children [30]. On the other hand, this bacterium was found to be underrepresented after sleeve gastrectomy surgery [31].

Of the three miRNAs associated with the abundance of *B. eggerthii* and BMI levels, miR-185-5p and miR-21-5p were also correlated with *D. longicatena* abundance. Furthermore, miR-185-5p was described as involved in oxidative stress, obesity and DM in many studies (reviewed at [32]). Moreover, miR-185-5p was identified as a regulator of de novo cholesterol biosynthesis and low density lipoprotein uptake [21]. However, we could not find in the literature evidences of association between this miRNA and gut microbiota.

Regarding miR-21-5p, the 16S rRNA sequencing revealed significant differences in the composition of WT and miR-21^−/−^ intestinal microbiota in a dextran sodium sulphate (DSS)-induced colitis mouse model [33]. Moreover, commensal bacteria induced the expression of miR-21 in IECs, with implications in the regulation of intestinal epithelial permeability [13]. Otherwise, miR-130b-3p was only correlated with *B. eggerthi* abundance and there is evidence that the expression of this miRNA was influenced by microbial status in intestinal epithelial stem cell of conventionalized mice compared to germ-free mice [14], showing that the miRNAs expression could be modulated by gut microbiota.

Moreover, an association among *B. eggerthi* abundance, miR-183-5p expression and adiponectin levels was also found. Previous findings demonstrated that miR-183 may contribute to adipocyte differentiation, adipogenesis and development of fat cells [22, 34]. Additionally, miR-183 was identified as a novel positive regulator during 3T3-L1 adipogenesis. Both gain-of-function and loss-of-function assays showed that miR-183 promoted 3T3-L1 adipocyte differentiation, lipid accumulation and adipogenesis by enhancing the expressions of peroxisome proliferator activated receptor gamma (PPARγ), CCAAT enhancer binding protein alpha (C/EBPα), adiponectin and fatty acid synthase (FAS) [35].

The expression of miR-15a-5p was found associated with *H. parainfluenzae* abundance and insulin levels in our study. miR-15a positively regulates insulin biosynthesis by inhibiting endogenous uncoupling protein 2 (*UCP2*) gene expression, leading to higher ATP levels in islets and improving glucose-stimulated insulin secretion. Moreover, circulating levels of miR-15a were found downregulated before the onset of type 2 DM (T2DM) [36] and also in incident-T2DM subjects compared to controls, with intermediate values in the pre-DM and incident pre-DM patients [37].

Regarding gut microbiota composition, our results evidenced that obesity had no significant impact in alpha diversity, indicating that microbial species diversity is relatively stable in response to obesity. However, obesity influenced the beta diversity of human gut microbiota compared to the control group, suggesting that this disease is accompanied by species replacement (changes in species taxa) and species sorting (changes in abundance).

According to a meta-analysis of metagenomic datasets obtained from fecal samples of healthy human adults living in different world regions, *Bacteroides* and *Barnesiella* genera are markers of Western populations [38]. *Barnesiella* spp. (represented mainly by the specie *Barnesiella intestinihominis*) were identified only in populations living in developed countries, suggesting that their presence was promoted by the urbanization/industrialization process and a Western-type diet [38].

In agreement with our results, the levels of *Dorea* genera were previously reported to be higher in overweight children compared to their normal weight counterparts [39]. Moreover, this association was stronger for non-white children than for white children and also stronger for boys than for girls [39]. Interestingly, a recent study in an early-life HFD mouse model found that the this diet increased the relative abundances of *Dorea* genus [40].

Our investigation has strengths and limitations. The strengths include study and data analyses of a very-well characterized cohort of subjects with obesity and eutrophic subjects was analyzed. Moreover, several quality controls for miRNA extraction, cDNA synthesis and PCR process were implemented. Additionally, robust bioinformatic analyses were performed to explore the pathways where these miRNAs target genes are participating, explaining the association with obesity. Likewise, we highlighted candidates for potentially linking host miRNAs and gut microbiota, which can be directly validated and explored in model systems.

Even though these methods are powerful, this evaluation has some limitations. First, the reduced sample size which could lead to lack of power to detect small differences in miRNA expression between groups and the absence of a validation cohort. First, it is important to note that our study uses 16S rRNA gene sequencing to characterize microbiome taxonomic composition. Second, the results from bioinformatics are predictions and may not represent the real biological system. Third, our approach identifies correlations and not causal relationships. Even though a hypothesis-driven approach was performed, selection of only miRNAs previously associated with obesity or metabolism makes possible type I or type II errors due to multiple comparisons. These limitations should be considered when interpreting the results. Although limitations exist in the current data, the patterns uncovered here are important for understanding the contribution of miRNAs and gut microbiota in obesity.

## 4. Materials and Methods

### 4.1. Study Population

This study was designed in accordance with STROBE guidelines for reporting association studies [41,42]. The sample comprised 78 subjects with obesity (cases, body mass index (BMI): 30–40 kg/m^2^) and 25 eutrophic individuals (controls, BMI ≤ 25 kg/m^2^). Obesity was classified following the World Health Organization (WHO) guidelines [2]. All volunteers were enrolled between October 2015 and February 2016 in the Metabolic Unit of the Centre for Nutrition Research of the University of Navarra, Spain. Major exclusion criteria included history of diabetes mellitus (DM), cardiovascular disease or hypertension, pregnant or lactating women, current use of lipid-lowering drugs or medications that affect body weight and weight change ≥3 kg within three months before the recruitment. All subjects were self-defined as Caucasians and all samples were collected in the morning, after 12 h fasting.

This study followed the ethical principles for medical research in humans from the Helsinki Declaration [43]. Moreover, the research protocol was properly approved by the Research Ethics Committee of the University of Navarra, Spain (ref. 132/2015) and it is registered at ClinicalTrials.gov (reg. no. NCT02737267). A written informed consent of each participant was obtained prior to enrollment in the study.

All patients underwent anthropometric and laboratory evaluations, as previously described [30,44]. The measurements of height (cm), body weight (kg) and waist circumference (WC, cm) were collected in the fasting state by trained nutritionists following validated procedures [30]. BMI was calculated as the ratio between weight and squared height (kg/m^2^). Body composition was quantified by dual-energy X-ray absorptiometry according to instructions provided by the supplier (Lunar Prodigy, software version 6.0, Madison, WI, USA). Biochemical measurements including fasting plasma glucose (FPG, mg/dL), total cholesterol (TC, mg/dL), high-density lipoprotein cholesterol (HDL-c, mg/dL) and triglycerides (TG, mg/dL) were determined in an automatic analyzer (Pentra C200, HORIBA Medical, Kyoto, Japan), following standardized procedures. Endocrine markers such as insulin, adiponectin and leptin were quantified with commercial ELISA kits (Mercodia Insulin ELISA, Biovendor Human adiponectin ELISA and Mercodia Leptin; Mercodia, Uppsala, Sweden).

Insulin resistance was estimated by the homeostatic model assessment-insulin resistance (HOMA-IR) index according to the following formula: (fasting insulin (mU/L) × plasma glucose (mmol/L)/22.5), while the triglyceride-glucose (TyG) index was calculated as: ln (fasting triglycerides (mg/dL) × fasting plasma glucose (mg/dL)/2), as described elsewhere [45].

A validated semiquantitative food frequency questionnaire was used to evaluate habitual consumption (daily, weekly, monthly or never) of 137 foods during the previous year [46]. Energy and nutrient intakes were further calculated with an ad hoc computer program based on the standard Spanish food composition tables [47]. The physical activity level was estimated using a validated questionnaire [48]. The volume of activity was expressed in metabolic equivalents (METs, kcal/kg/h), as described elsewhere [49].

### 4.2. MicroRNA Expression Analysis

#### 4.2.1. microRNA Isolation and Reverse Transcription-Quantitative PCR

Total RNA was extracted from 200 µL EDTA-plasma using the miRNeasy Serum/Plasma Advanced kit (Qiagen, Hilden, Germany) according to the manufacturer’s recommendations. RNA spike-in was added to each sample (RNA Spike-In Kit, Qiagen, Hilden, Germany). The purity of RNA samples was assessed by analyzing RNA Spike-In expression.

Relative expression of the 86 miRNAs was analyzed in plasma from all subjects using the Custom Pick-&-Mix microRNA PCR Panel v5 (Qiagen, Hilden, Germany). Moreover, 9 controls (reference genes + Spike-in controls) and a blank were also included in each plate, as shown in Table S1. The selection of these miRNAs was based on the available literature [9,26] and by searching the miRWalk 2.0 database [50] for those miRNAs potentially associated with obesity in humans.

Total RNA (4 µL) was reverse-transcribed in 10 µL reaction using the miRCURY LNA Universal RT microRNA PCR, Polyadenylation and cDNA synthesis kit II (Qiagen, Hilden, Germany). RT-qPCR experiments were performed using a CFX384 Real-time system (Bio-Rad, Hercules, CA, USA). The cycling conditions were: 95 °C for 2 min, followed by 40 cycles at 95 °C for 10 s and 56 °C for 1 min. Relative expressions were calculated using the 2^∆∆Cq^ method [51].

#### 4.2.2. Quality Control and Normalization

Quality control was carried out using synthetic spike-in RNAs to analyze the robustness of RNA isolation process and quality of extracted miRNA. The RNA isolation controls (UniSp2, UniSp4 and UniSp5; Qiagen, Hilden, Germany) were added to the thawed plasma before the isolation process, aiming to detect differences in extraction efficiency. The cDNA synthesis control (UniSp6, Qiagen, Hilden, Germany) and cel-miR-39-3p were added to the reverse transcription reaction to determine the effectiveness of this process. Furthermore, UniSp3 was included in all plates and used as an inter-plate calibrator and PCR amplification control.

Hemolysis was assessed by the ratio between hsa-miR-451a (which is expressed in erythrocytes) and hsa-miR-23a-3p (which is relatively stable in plasma and not affected by hemolysis) as described elsewhere [52]. The difference in expression values between these 2 miRNAs provides a good measure of the extent of hemolysis, with values > 5 suggesting erythrocyte miRNA contamination. Only samples without hemolysis (values < 5) were included in the study [52]. The assay cut-off was 35 cycles and miRNAs expressed in at least 20% of the total sample [53]. All individual samples were run on a predefined assay panel of 96 specific human miRNAs (Table S1). The miRNAs with complete data were used for the global mean method for normalization of the data, since this approach was found to be the most stable normalizer [54].

#### 4.2.3. miRNA Target Prediction and Pathway Enrichment Analysis

Potential targets of selected miRNAs were searched using miRWalk 3.0 (http://zmf.umm.uni-heidelberg.de/apps/zmf/mirwalk2/, accessed on 4 August 2020). To better understand the biological relevance of the miRNAs target genes, a functional enrichment analysis was executed using PathDIP (accessed on 4 August 2020, [55]). A hypergeometric test was used to calculate the statistical significance of the enriched pathways and *p*-values were corrected for multiple tests using the Benjamini-Hochberg procedure, which provides a False Discovery Rate (FDR) adjusted-*p*-value (*q*-value). Pathways associated with a *q*-value <0.05 were considered significantly enriched.

### 4.3. Gut Microbiota Analysis

#### 4.3.1. Fecal Sample Collection and DNA Extraction

Volunteers self-collected fecal samples using OMNIgene•GUT kits from DNA Genotek (Ottawa, ON, Canada), according to the standardized instructions provided by the fabricant. The isolation of DNA from fecal samples was performed with the QIAamp^®^ DNA kit (Qiagen, Hilden, Germany) following the manufacturer’s protocol.

#### 4.3.2. 16S rRNA Sequencing and Sequence Analysis

Bacterial DNA sequencing was performed by the *Servei de Genòmica i Bioinformàtica* from the Autonomous University of Barcelona (Barcelona, Spain). The Illumina 16S protocol, based on the amplification of the V3-V4 variable regions of the 16S rRNA gene, was followed. Paired-end sequencing was performed on the MiSeq System (Illumina, San Diego, CA, USA). For this methodology, two PCR reactions were carried out. In the first one, 12.5 ng of genomic DNA and 16S-F and 16S-R primers were used. After the cleaning process, 5 μL of the first PCR was used in the second PCR. The primers used in this PCR were part of the Nextera XT DNA Index Kit (96 indexes, 384 samples) FC-131-1002 (Illumina, San Diego, CA, USA). After each PCR, the quality of the process was checked in a Labchip Bioanalyzer (Agilent Technologies Inc., Santa Clara, CA, USA). Once all the samples were obtained, up to 40 samples were multiplexed in each run of 2 × 300 cycles for obtaining around 500,000 reads per sample. For this purpose, equimolar concentrations of each of the samples were mixed and the pool was diluted up to 20 pM. A total of 3 runs were performed on the MiSeq sequencer with the MiSeq Reagent Kit v3 (600 cycle) MS-102-3003. The maximum of reads obtained was 1,867,496 and the minimum 5019; the mean was 164,387.

The 16S rRNA sequences were filtered following the quality criteria of the OTU processing pipeline LotuS (release 1.58) [56]. This pipeline includes UPARSE de novo sequence clustering [57] and the removal of chimeric sequences and phix contaminants for the identification of Operational Taxonomic Units (OTUs) and their abundance matrix generation [58,59]. OTU refers to organisms clustered by similarities in DNA sequence [60]. Finally, taxonomy was assigned using BLAST [61] and HITdb [62], achieving up to species-level sensitivity. The abundance matrices were first filtered and then normalized in R/Bioconductor [63,64] at each classification level: OTUs, phylum, genus, family, order, class and species. Briefly, taxa less than 10% of frequency in our population were removed for the analysis and a global normalization was performed using the library size as a correcting factor and log2 data transformation.

To evaluate alpha diversity, the Shannon index was calculated [65]. To assess beta diversity, permutational multivariate analysis of variance (PERMANOVA) was used to analyze whether the structures of gut microbiota were significantly different among groups based on the Jaccard and Bray–Curtis distance matrices [66].

### 4.4. Statistical Analysis

Normalized data (RQ expression levels) were initially analyzed, with an estimation and comparison of expression levels between groups. Normal distribution of data was assessed using the Kolmogorov-Smirnov and Shapiro-Wilk tests. Variables with normal distribution are presented as mean ± standard deviation (SD). Variables with skewed distribution were log-transformed prior to analysis and are presented as median (25th–75th percentiles). Categorical data are shown as percentages. Clinical and laboratory characteristics, miRNA expressions and gut microbiota abundance were compared among groups using Student’s *t*-test or χ^2^ tests, as appropriate. Correlations between quantitative variables were assessed using Pearson’s correlation tests. The two-way ANOVA full factorial test was used in our study to identify relations between miRNA expression, microbiota and laboratorial characteristics. Linear regression was used to identify factors that might influence miRNA expression or microbiome abundance, including age, gender, triglycerides and HOMA-IR. Multivariate regression models were applied to find potential covariates (Model 1: age and gender; Model 2: gender, triglycerides and HOMA-IR).

All classical statistical analyses were performed using the SPSS statistical package (v.20.0) for Windows (SPSS Inc., Chicago, IL, USA) and PAST v3.24 (University of Oslo, Norway) were used for statistical analyses of biodiversity. Raw *p*-values were adjusted by the Benjamini-Hochberg’s FDR controlling procedure (*q*-value < 0.05). The network visualization of miRNA-variables was generated using Cytoscape v.3.7.1 [67]. One heatmap plot of the correlation values were produced using MORPHEUS web tool (Morpheus, Cambridge, MA, USA. https://software.broadinstitute.org/morpheus, accessed on 4 August 2020). Graphics were performed using Ploty chart studio (https://chart-studio.plotly.com, accessed on 4 August 2020).

## 5. Conclusions

This current research characterized a global relationship between microbial community composition and miRNA expression in plasma of subjects with obesity compared to normal weight individuals. Indeed, our study featured an interaction between *B. eggerthi* abundance and circulating miRNA expression in the control of body adiposity. The current study also adds to the growing body of literature that suggests that miRNAs may serve as a communication mechanism between the gut microbiota and human hosts.

## Figures and Tables

**Table 1:** Clinical, dietary and laboratory characteristics of the sample included in the study.

Characteristics	Subjects with Obesity (Cases, *n* = 78)	Eutrophic Individuals (Controls, *n* = 25)	*p*-Value *
Age (years)	46.6 ± 9.4	44.7 ± 9.1	0.106
Gender (% male)	36.1	40.0	0.443
Smoking habits (%)			
Ex-smokers	41.7	33.4	0.251
Smokers	25.0	20.8	
Non-smokers	44.0	45.8	
Alcohol consumption			
Yes	51.4	64.0	0.063
No	48.6	36.0	
Anthropometric and clinical data			
BMI (kg/m^2^)	32.9 ± 2.4	18.6 ± 2.1	
WC (cm)	104.9 ± 10.2	75.2 ± 7.6	0.0001
SBP (mmHg)	131 ± 16	111 ± 10	0.0001
DBP (mmHg)	86 ± 9	70 ± 8	0.0001
Metabolic profile			
FPG (mg/dL)	97.4 ± 11.9	85.3 ± 6.8	0.0001
TC (mg/dL)	222.5 ± 40.1	192.6 ± 37.1	0.001
HDL-c (mg/dL)	54.2 ± 14.0	61.6 ± 12.7	0.022
TG (mg/dL)	101.6 ± 54.1	65.6 ± 25.0	0.002
TyG index	4.6 ± 0.3	4.2 ± 0.2	0.0001
HOMA-IR index	1.6 (1.1–2.8)	0.6 (0.4–1.0)	0.0001
Adiponectin (ng/mL)	10.9 (7.9–13.5)	12.2 (9.3–15.7)	0.067
Insulin (mU/L)	6.8 (4.7–11.5)	3.2 (2.8–4.8)	0.0001
Leptin (ng/mL)	33.1 (17.2–46.8)	4.9 (2.1–11.7)	0.0001
Body composition			
Fat mass (%)	34.7 ± 6.5	13.6 ± 5.7	0.0001
Lean mass (%)	57.0 ± 11.7	47.6 ± 12.2	0.001
Dietary intake and energy expenditure			
Energy (Kcal)	2961 ± 1051	2588 ± 701	0.101
Carbohydrates (%)	41.4 ± 7.1	44.8 ± 6.4	0.034
Protein (%)	16.7 ± 2.9	15.9 ± 3.4	0.245
Fat (%)	40.1 ± 6.4	37.7 ± 4.9	0.100
Fiber (g/day)	27.9 ± 11.4	32.7 ± 11.5	0.070
METs (kcal/kg/h)	17.0 (7.5–27.0)	33.2 (20.0–44.4)	0.001

Variables are shown as mean ± SD, median (25th–75th percentiles) or %, as appropriate. * *p*-values were computed using χ^2^ or Student’s *t*-test, as appropriated. BMI: body mass index; DBP: diastolic blood pressure; FPG: fasting plasma glucose; HDL-c: high-density lipoprotein cholesterol; HOMA-IR index: homeostatic model assessment-insulin resistance index; METs: metabolic equivalents; SBP: systolic blood pressure; TC: total cholesterol; TG: triglycerides; TyG index: triglyceride glucose index; WC: waist circumference.

**Table 2:** Relation of 26 microRNAs whose expression profile in plasma is significantly different between cases with obesity and eutrophic controls.

microRNA	Subjects with Obesity (Cases, *n* = 78)	Eutrophic Individuals (Controls, *n* = 25)	*p*-Value *	*q*-Value **	Model 1 (*q*-Value)	Model 2 (*q*-Value)
miR-103a-3p	0.169 (0.057–0.443)	0.567 (0.263–1.426)	0.006	0.020	0.011	0.029
miR-107	0.201 (0.067–0.520)	0.614 (0.309–1.386)	0.014	0.038	0.022	0.044
miR-130a-3p	19.321 (6.029–32.947)	47.904 (31.421–79.317)	0.005	0.004	0.003	0.009
miR-130b-3p	0.208 (0.096–0.433)	0.442 (0.221–1.426)	0.003	0.015	0.011	0.049
miR-140-3p	0.237 (0.076–0.578)	0.872 (0.548–1.561)	0.0001	0.0012	0.001	0.006
miR-142-5p	0.118 (0.036–0.214)	0.279 (0.162–0.941)	0.002	0.007	0.004	0.020
miR-144-3p	1.308 (0.276–2.872)	6.903 (2.036–11.542)	0.0001	0.0012	0.001	0.009
miR-148a-3p	0.259 (0.096–0.479)	0.647 (0.212–1.135)	0.006	0.019	0.010	0.010
miR-181a-5p	0.549 (0.229–1.107)	1.633 (0.369–3.021)	0.006	0.008	0.007	0.006
miR-183-5p	0.374 (0.244–0.649)	0.775 (0.476–1.928)	0.001	0.009	0.002	0.026
miR-185-5p	0.198 (0.083–0.480)	0.665 (0.288–1.272)	0.034	0.040	0.029	0.036
miR-200c-3p	0.540 (0.257–1.118)	1.001 (0.454–2.161)	0.037	0.044	0.041	0.039
miR-205-5p	0.163 (0.122–0.559)	0.711 (0.319–1.322)	0.005	0.020	0.008	0.031
miR-21-5p	0.257 (0.137–0.615)	0.559 (0.261–1.238)	0.036	0.041	0.031	0.032
miR-210-3p	0.110 (0.071–0.245)	0.503 (0.117–0.672)	0.030	0.048	0.032	0.010
miR-221-3p	0.212 (0.065–0.454)	0.555 (0.268–1.549)	0.004	0.013	0.009	0.010
miR-222-3p	0.311 (0.158–0.712)	0.859 (0.470–1.396)	0.005	0.019	0.011	0.009
miR-15a-5p	0.118 (0.060–0.322)	0.356 (0.155–0.877)	0.022	0.054	0.019	0.020
miR-22-3p	0.126 (0.056–0.286)	0.320 (0.127–1.027)	0.012	0.034	0.019	0.099
miR-29c-3p	0.137 (0.068–0.350)	0.453 (0.202–1.269)	0.007	0.020	0.009	0.040
miR-30a-5p	0.629 (0.294–1.347)	1.426 (0.515–2.062)	0.043	0.048	0.044	0.034
miR-30c-5p	0.274 (0.093–0.625)	0.694 (0.235–1.462)	0.042	0.050	0.041	0.054
miR-33a-5p	1.268 (0.573–2.425)	4.54 (1.009–23.596)	0.012	0.016	0.029	0.048
miR-375	0.228 (0.124–0.609)	0.765 (0.484–1.563)	0.0001	0.002	0.001	0.009
miR-424-3p	0.892 (0.698–1.135)	2.000 (0.834–3.127)	0.016	0.030	0.034	0.076
miR-486-3p	0.301 (0.190–0.570)	0.645 (0.238–1.326)	0.004	0.009	0.011	0.040

Data are shown as median (25th–75th percentiles) of n-fold values. * *p*-values were obtained using Student *t*-test with the log-transformed variable. ** Raw *p*-values were corrected by false discovery rate (FDR; *q*-value). Regression analysis were done to compute a) model 1 (adjustment for age and gender) and b) model 2 (adjustment for age, gender, triglycerides and HOMA-IR). Statistical significance is shown as *q*-value < 0.05.

**Table 3:** Bacterial species whose abundance is statistically different between cases with obesity and eutrophic controls.

Bacteria	Subjects with Obesity (Cases, *n* = 78)	Eutrophic Individuals (Controls, *n* = 25)	*p*-Value *	*q*-Value **	Model 1 (*q*-Value)	Model 2 (*q*-Value)
*Abiotrophia defectiva*	0.0001 (0.0001–0.2825)	0.0001 (0.0001–0.001)	0.0001	0.001		
*Actinomyces odontolyticus*	0.0001 (0.0001–1.643)	0.0001 (0.0001–0.652)	0.010	0.020	0.037	0.047
*Allisonella histaminiformans*	0.0001 (0.0001–5.260)	0.0001 (0.0001–0.001)	0.0001	0.001	0.026	0.042
*Bacteroides eggerthii*	3.573 (1.903–10.086)	9.769 (3.32–11.892)	0.039	0.042	0.049	0.049
*Barnesiella intestinihominis*	2.885 (0.730–5.809)	1.889 (0.001–3.361)	0.019	0.036	0.047	0.047
*Dorea longicatena*	9.959 (9.295–10.557)	9.547 (8.887–10.268)	0.039	0.045	0.049	0.049
*Haemophilus parainfluenzae*	5.834 (2.990–7.775)	8.254 (6.181–10.708)	0.001	0.002	0.003	0.032
*Howardella ureilytica*	2.439 (0.0001–6.303)	0.0001 (0.0001–2.476)	0.010	0.023	0.038	0.037
*Lactobacillus curvatus*	0.0001 (0.0001–1.626)	0.0001 (0.0001–0.001)	0.0001	0.001	0.046	0.050
*Megamonas funiformis*	0.0001 (0.0001–0.974)	0.0001 (0.0001–0.001)	0.0001	0.001	0.050	0.143
*Mitsuokella jaladudinii*	0.0001 (0.0001–2.521)	0.0001 (0.0001–0.001)	0.002	0.030		
*Odoribacter laneus*	0.0001 (0.0001–2.707)	0.0001 (0.0001–1.015)	0.027	0.040	0.291	0.605

Data are shown as median (25th–75th percentiles). * *p*-values were obtained using Student *t*-test with the log-transformed variable. ** Raw *p*-values were corrected by false discovery rate (FDR; *q*-value). Regression analysis were done to compute (i) model 1 (adjustment for age and gender) and (ii) model 2 (adjustment for age, gender, triglycerides and HOMA-IR). Statistical significance is shown as *q*-value < 0.05.

## Supplementary Materials

The following are available online at https://www.mdpi.com/1422-0067/21/24/9509/s1, Table S1. Assays reference numbers of the 96 miRNAs included in the Pick-&-Mix Custom plate. Table S2. Regression models for microRNA expression between subjects with obesity and eutrophic individuals. Table S3. Regression models for gut microbiota abundance between subjects with obesity and eutrophic individuals. Table S4. Target genes of the 14 miRNAs correlated with bacterial species associated with obesity. Table S5. KEGG pathways in which the target genes of the 14 miRNAs participate. Figure S1. Gut microbiota alpha diversity using Shannon index. Figure S2. Spatial variation of the beta diversity based on principal coordinates analysis (PCoA) using (A) Jaccard and (B) Bray-Curtis dissimilarities. Beta diversity was tested by permutation multivariate analysis of variance (PERMANOVA; *p* < 0.05). Figure S3. Validated target genes of 14 miRNAs correlated with bacterial species associated with obesity, according to miRTarBase, downloaded from miRWalk database. This network figure was done using Cytoscape v.3.7.1.
