# Supplementary material for: Comprehensive Analysis Reveals Novel Interactions between Circulating MicroRNAs and Gut Microbiota Composition in Human Obesity
Source: Int J Mol Sci. 2020 Dec 14;21(24):9509. doi: 10.3390/ijms21249509 (PMC7765005; doi:10.3390/ijms21249509)
Supplement: Supplementary file 1 [file ijms-21-09509-s001.zip › Supplementary files/Table S3.docx]

**Supplementary table 3.** Regression models for gut microbiota abundance between subjects with obesity and eutrophic individuals.

| **Bacteria** | **Model 1** | **Model 2** |
| --- | --- | --- |
| Abiotrophia defectiva | - | - |
| Actinomyces odontolyticus | 1.820 (0.959 – 3.454); 0.067 | 1.542 (0.769 – 3.094); 0.233 |
| **Allisonella histaminiformans** | **1.379 (1.040 -1.829); 0.026** | **1.398 (1.013 – 1.929); 0.042** |
| **Bacteroides eggerthii** | **0.899 (0.806 – 0.998); 0.049** | **0.917 (0.808 – 0.998); 0.049** |
| **Barnesiella intestinihominis** | **1.245 (1.003 – 1.546); 0.047** | **1.195 (1.001 – 1.520); 0.047** |
| **Dorea longicatena** | **0.649 (0.419 – 0.996); 0.049** | **0.772 (0.480 – 0.998); 0.049** |
| **Haemophilus parainfluenzae** | **0.749 (0.619 – 0.906); 0.003** | **0.793 (0.641 – 0.980); 0.032** |
| **Howardella ureilytica** | **1.212 (1.011 – 1.453); 0.038** | **1.249 (1.013 – 1.538); 0.037** |
| Lactobacillus curvatus | 2.358 (1.016 – 5.471); 0.046 | 2.330 (0.925 – 5.873); 0.073 |
| Megamonas funiformis | 3.879 (0.613 – 24.551); 0.150 | 7.123 (0.5123 – 16.563); 0.143 |
| Mitsuokella jaladudinii | - | - |
| Odoribacter laneus | 1.337 (0.780 – 2.291); 0.291 | 1.187 (0.621 – 2.265); 0.605 |

Data are shown as median (25th–75th percentiles) of n-fold values. *P values were obtained using Student t test using the log-transformed variable.

MODEL 1: Adjustment for age and gender.

MODEL 2: Adjustment for age, gender, triglycerides, and HOMA-IR.
