# Supplementary material for: Comprehensive Analysis Reveals Novel Interactions between Circulating MicroRNAs and Gut Microbiota Composition in Human Obesity
Source: Int J Mol Sci. 2020 Dec 14;21(24):9509. doi: 10.3390/ijms21249509 (PMC7765005; doi:10.3390/ijms21249509)
Supplement: Supplementary file 1 [file ijms-21-09509-s001.zip › Supplementary files/Table S2.docx]

**Supplementary table 2.** Regression models for microRNA expression between subjects with obesity and eutrophic individuals.

| **microRNA** | **Model 1** | **Model 2** |
| --- | --- | --- |
| miR-103a-3p | 0.342 (0.149 – 0.784); 0.011 | 0.331 (0.123 – 0.893); 0.029 |
| miR-107 | 0.341 (0.136 – 0.857); 0.022 | 0.329 (0.112 – 0.970); 0.044 |
| miR-130a-3p | 0.195 (0.065 – 0.583); 0.003 | 0.133 (0.030 – 0.603); 0.009 |
| miR-130b-3p | 0.246 (0.084 – 0.722); 0.011 | 0.152 (0.039 – 0.598); 0.050 |
| miR-140-3p | 0.069 (0.015 – 0.327); 0.001 | 0.065 (0.009 – 0.451); 0.006 |
| miR-142-5p | 0.208 (0.071 – 0.607); 0.004 | 0.111 (0.027 – 0.713); 0.020 |
| miR-144-3p | 0.140 (0.043 – 0.461); 0.001 | 0.045 (0.004 – 0.465); 0.009 |
| miR-148a-3p | 0.197 (0.057 – 0.680); 0.010 | 0.057 (0.007 – 0.500); 0.010 |
| miR-181a-5p | 0.242 (0.086 – 0.678); 0.007 | 0.129 (0.030 – 0.554); 0.006 |
| miR-183-5p | 0.052 (0.008 – 0.353); 0.002 | 0.082 (0.009 – 0.738); 0.026 |
| miR-185-5p | 0.415 (0.189 – 0.912); 0.029 | 0.297 (0.110 – 0.797); 0.016 |
| miR-200c-3p | 0.232 (0.057 – 0.945); 0.041 | 0.162 (0.029 – 0.910); 0.039 |
| miR-205-5p | 0.150 (0.037 – 0.606); 0.008 | 0.051 (0.007 – 0.374); 0.003 |
| miR-21-5p | 0.320 (0.114 – 0.900); 0.031 | 0.234 (0.062 – 0.884); 0.032 |
| miR-210-3p | 0.305 (0.103 – 0.905); 0.032 | 0.113 (0.022 – 0.591); 0.010 |
| miR-221-3p | 0.242 (0.084 – 0.698); 0.009 | 0.113 (0.022 – 0.598); 0.010 |
| miR-222-3p | 0.213 (0.064 – 0.705); 0.011 | 0.084 (0.013 – 0.532); 0.009 |
| miR-15a-5p | 0.356 (0.150 – 0.843); 0.019 | 0.291 (0.103 – 0.820); 0.020 |
| miR-22-3p | 0.325 (0.127 – 0.829); 0.019 | 0.381 (0.121 – 1.200); 0.099 |
| miR-29c-3p | 0.274 (0.103 – 0.728); 0.009 | 0.261 (0.073 – 0.938); 0.040 |
| miR-30a-5p | 0.294 (0.089 – 0.967); 0.044 | 0.166 (0.032 – 0.869); 0.034 |
| miR-30c-5p | 0.465 (0.223 – 0.968); 0.041 | 0.407 (0.164 – 1.014); 0.054 |
| miR-33a-5p | 0.359 (0.143 – 0.901); 0.029 | 0.169 (0.043 – 0.930); 0.048 |
| miR-375 | 0.057 (0.011 – 0.287); 0.001 | 0.051 (0.005 – 0.480); 0.009 |
| miR-424-3p | 0.069 (0.006 – 0.820); 0.034 | 0.027 (0.001 – 2.443); 0.116 |
| miR-486-3p | 0.159 (0.038 – 0.658); 0.011 | 0.153 (0.023 – 1.031); 0.054 |

Data are shown as median (25th–75th percentiles) of n-fold values. *P values were obtained using Student t test using the log-transformed variable.

MODEL 1: Adjustment for age and gender.

MODEL 2: Adjustment for age, gender, triglycerides, and HOMA-IR.
