# Supplementary figures and images for: Comprehensive Analysis Reveals Novel Interactions between Circulating MicroRNAs and Gut Microbiota Composition in Human Obesity
Source: Int J Mol Sci. 2020 Dec 14;21(24):9509. doi: 10.3390/ijms21249509 (PMC7765005; doi:10.3390/ijms21249509)

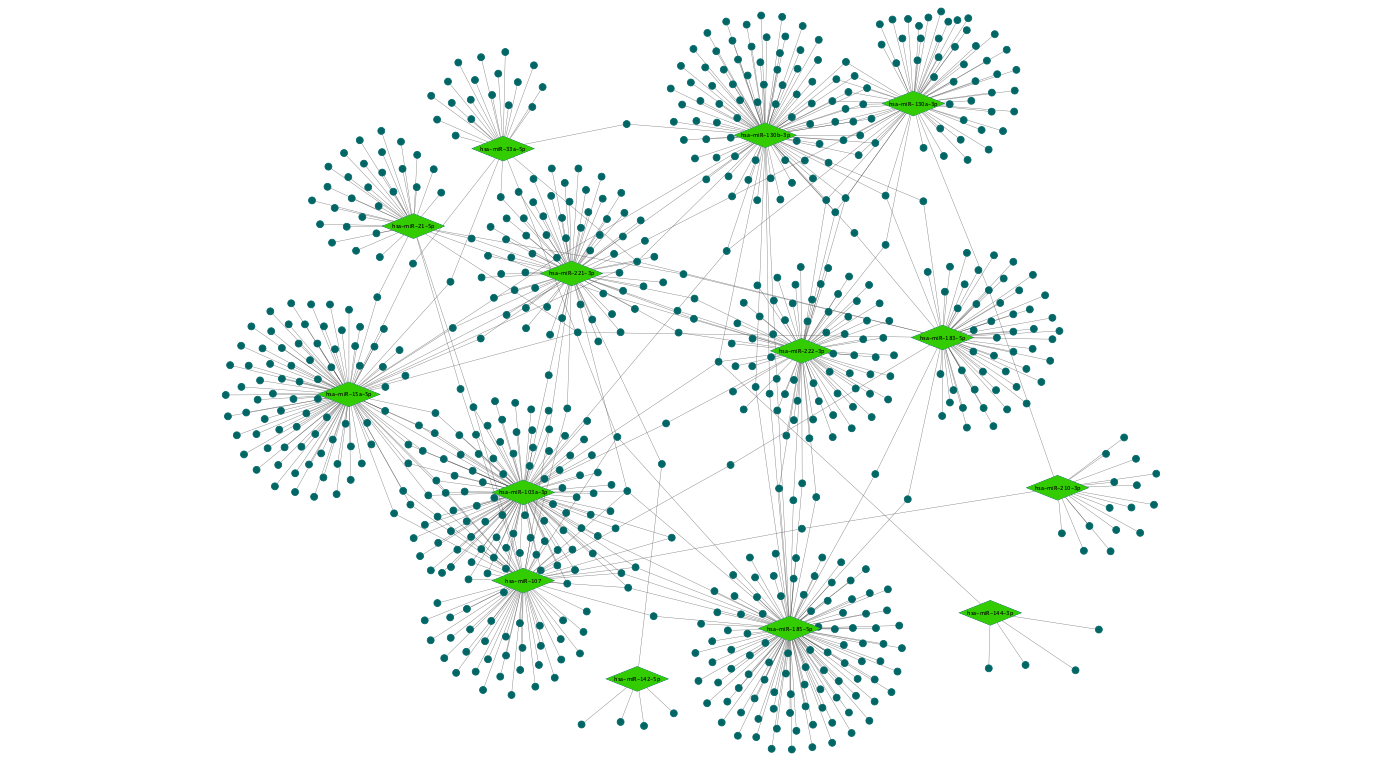

Supplement: Supplementary file 1 [file ijms-21-09509-s001.zip › Supplementary files/Figure S3.png]

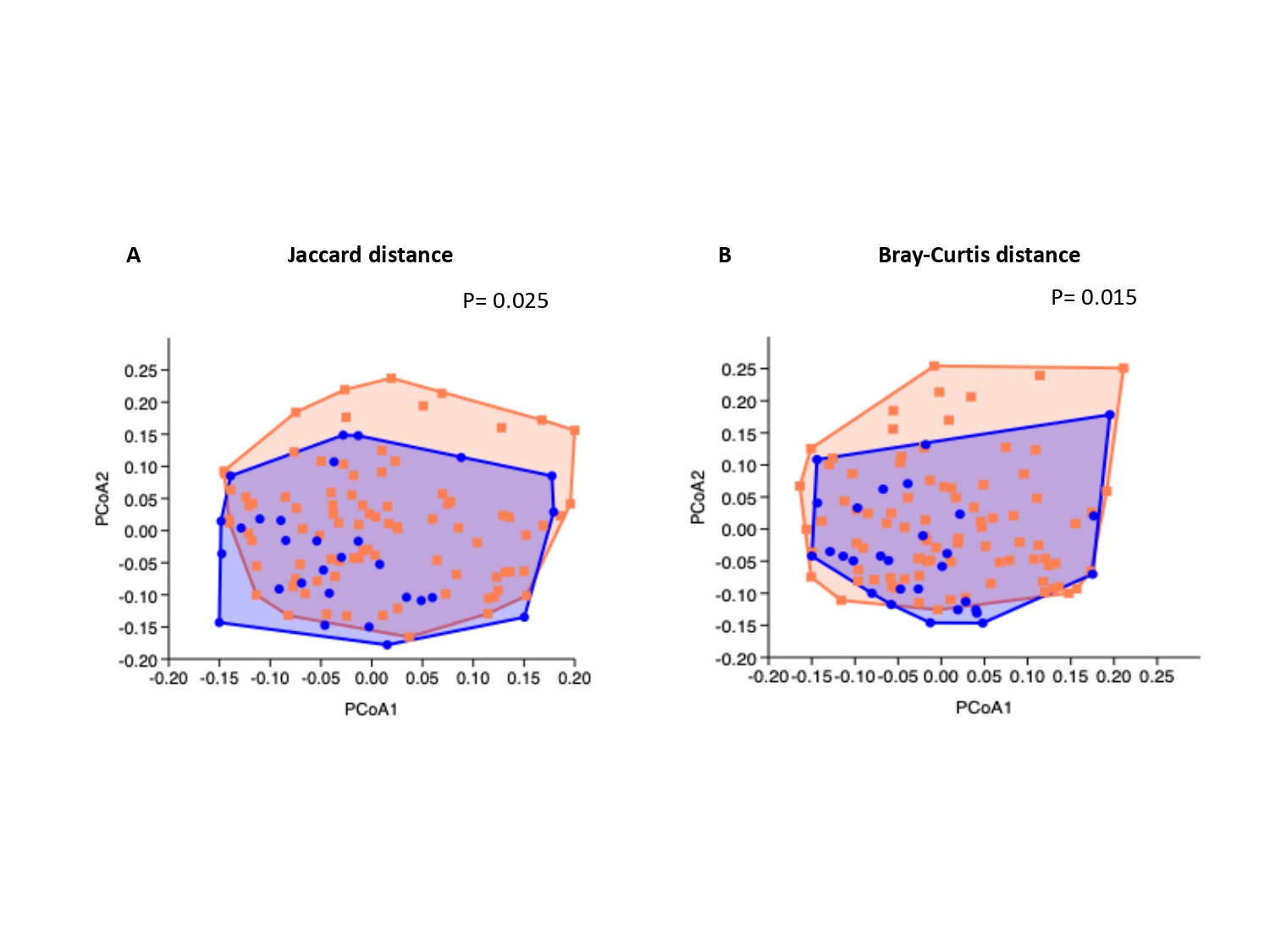

Supplement: Supplementary file 1 [file ijms-21-09509-s001.zip › Supplementary files/Figure S2.jpg]

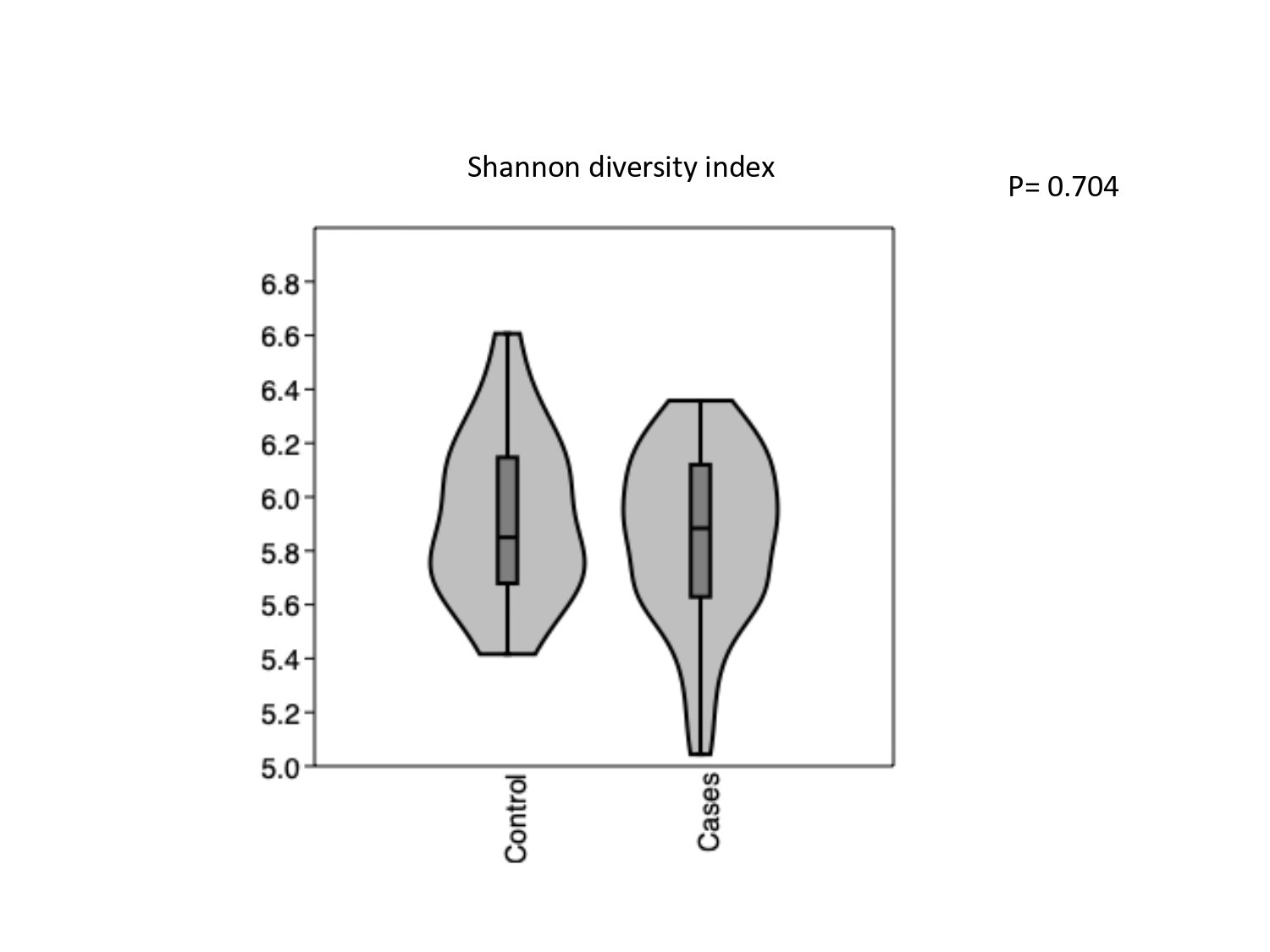

Supplement: Supplementary file 1 [file ijms-21-09509-s001.zip › Supplementary files/Figure S1.jpg]
